# From Design to Screening: A New Antimicrobial Peptide Discovery Pipeline

**DOI:** 10.1371/journal.pone.0059305

**Published:** 2013-03-19

**Authors:** Saadet Albayrak Guralp, Yusuf E. Murgha, Jean-Marie Rouillard, Erdogan Gulari

**Affiliations:** 1 Department of Chemical Engineering, University of Michigan, Ann Arbor, Michigan, United States of America; 2 Mycroarray, Ann Arbor, Michigan, United States of America; Naval Research Laboratory, United States of America

## Abstract

Antimicrobial peptides (AMPs) belong to a class of natural microbicidal molecules that have been receiving great attention for their lower propensity for inducing drug resistance, hence, their potential as alternative drugs to conventional antibiotics. By generating AMP libraries, one can study a large number of candidates for their activities simultaneously in a timely manner. Here, we describe a novel methodology where *in silico* designed AMP-encoding oligonucleotide libraries are cloned and expressed in a cellular host for rapid screening of active molecules. The combination of parallel oligonucleotide synthesis with microbial expression systems not only offers complete flexibility for sequence design but also allows for economical construction of very large peptide libraries. An application of this approach to discovery of novel AMPs has been demonstrated by constructing and screening a custom library of twelve thousand plantaricin-423 mutants in *Escherichia coli*. Analysis of selected clones by both Sanger-sequencing and 454 high-throughput sequencing produced a significant amount of data for positionally important residues of plantaricin-423 responsible for antimicrobial activity and, moreover, resulted in identification of many novel variants with enhanced specific activities against *Listeria innocua*. This approach allows for generation of fully tailored peptide collections in a very cost effective way and will have countless applications from discovery of novel AMPs to gaining fundamental understanding of their biological function and characteristics.

## Introduction

The rapid increase in antibiotic-resistant pathogenic bacteria is one of the main health problems of this century due to excessive and often inappropriate use of antibiotics in human and animal health care for the treatment and prevention of infections [1]. There is, consequently, an immediate need for the development of novel antimicrobial drugs with different mechanisms of action on target microorganism than that of existing antibiotics. Antimicrobial peptides (AMPs) play an important role as a first line of defense in every life form due to their broad spectrum native microbicidal activity and a range of immune-modulatory functions [2], [3]. AMPs show extreme diversity in their sequence, size, and structure, but they all share two functionally important properties: an overall positive charge and a high proportion of hydrophobic residues [4]. These peptides are active at nanomolar to micromolar concentrations and most of them kill their target microorganism via a non-receptor mediated mechanism involving permeation of the target membrane [5], [6].

A significant amount of research is currently focused on developing novel AMPs for therapeutic, biomedical, and biotechnological applications (see references [7], [8], [9], [10] for a few extensive reviews). Current methodologies used for the construction of AMP libraries present both advantages and disadvantages when it comes to sequence design, peptide length, or the library size. PCR-based techniques, such as site-saturation mutagenesis [11], [12] and DNA shuffling [13], where randomly-generated nucleic acid libraries encoding for AMPs are expressed in a biological host, offer large library complexity and the peptide length is not restricted in most systems. Since the mutations are introduced in a random fashion, however, the user control over sequence design is very limited in these techniques. Synthetic combinatorial methods, on the other hand, allow for custom sequence design and a variety of high-throughput screening assays, hence, they have been successfully employed for generating combinatorial AMP libraries [14], [15], [16]. However, these systems are still limited by the peptide length (optimum length up to 20 amino acids) as well as the library size due to intense labor and high cost associated with complex synthetic chemistry [17], [18].

The main goal of this study was to develop a platform that combines the design flexibility of synthetic methods with the ability of biological techniques for producing large libraries, which would enable researchers to study fully defined AMP libraries in a high-throughput and economical manner. We, hereby, describe a novel approach for the construction of large custom peptide libraries by combining light-directed *in situ* parallel oligonucleotide synthesis with a cellular expression and screening system. The parallel oligonucleotide synthesis technology allows for each entity of the library to be fully defined and is suitable for the maskless synthesis of large numbers of oligonucleotides on a single array in a very cost-effective way [19]. *In vivo* screening of peptide libraries have been successfully done in a variety of cellular expression hosts including *Escherichia coli* [20], *Lactococcus lactis* [21], and *Saccharomyces cerevisiae* [22]. Therefore, by using this strategy, libraries containing tens of thousands of custom-designed AMP candidates can be screened in a secretory expression host against any desired target organism at a much lower cost compared to synthetic libraries.

To demonstrate the feasibility of this method, we have constructed an AMP library encoding for twelve thousand plantaricin-423 mutants and screened it against gram-positive bacteria *Listeria innocua*. Plantaricin-423 (or Pln-423) is a 37-amino acid Class II-a bacteriocin produced by *Lactobacillus plantarum* 423 and it displays bactericidal activities against several foodborne pathogens and spoilage gram positive bacteria, hence, presents great potential to be used as a biopreservative [23]. Our findings in this study successfully demonstrate how synthetic oligonucleotide pools can be employed for the generation of custom peptide libraries and the discovery of novel variants with desired properties. Each step of the process is explained in detail below and the application of this method to study the Pln-423 mutant library is discussed in the following section.

## Results

### Description of the Method

**Figure 1:**
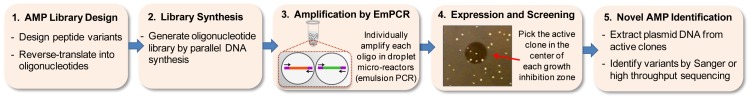
Diagram of the five-step process for the construction and screening of AMP libraries.

Construction of AMP-encoding libraries from oligonucleotide pools is a 5-step process (Figure 1); the first three steps and the last one are application-independent, however, the fourth step can be varied based on the choice of the expression host and the screening assay that are suitable for the library of interest.

#### Steps 1 and 2: Library design and synthesis

The peptide library can either be designed based on established guidelines and/or desired mutations can be systematically introduced to a peptide of interest. The maximum peptide size is limited by the length of oligonucleotides that can be efficiently synthesized, which is currently up to 200mer (with our light-directed synthesis technology) including two 20mer primer binding sites for amplification. Any other parallel DNA synthesis technology yielding libraries of long oligonucleotides is suitable as well. Following *in silico* peptide library generation (each peptide up to 50aa long), the amino acid sequences are reverse-translated into codon-optimized oligonucleotides by following two parameters: first, the most abundant codon of the host organism for each amino acid is selected for optimum expression [24], [25], [26]; second, in case the most abundant codon for a particular amino acid leads to a homopolymer formation in the sequence, then the second most abundant codon is used in that position to minimize errors during synthesis as well as amplification [27]. The oligonucleotide library is synthesized on glass slides using combination of standard phosphoramidite chemistry and maskless photolithography. After cleavage off the chips and purification, the library is ready for amplification.

#### Step 3: Amplification by emulsion PCR

The single-stranded oligonucleotides are amplified by PCR to generate sufficient amount of double-stranded DNA and to add restriction sites for the subsequent cloning experiments. To prevent cross-recombination events between the homologous regions of the template DNA fragments and reduce competition between fragments of different length, the amplification of the oligonucleotide libraries is performed by emulsion PCR (emPCR) [28], [29]. Single oligonucleotide molecules are individually and independently amplified in micro-droplets formed by the emulsification of the PCR reaction mixture in oil. This method enables equal-representation of each DNA fragment and reduces the formation of artifactual molecules, as often seen in conventional PCR, thus preserving library complexity [29], [30].

#### Step 4: Library expression in escherichia coli and activity screening

For the *in vivo* production of AMPs, we have employed an *E. coli* expression system [31] that relies on periplasmic-expression of recombinant peptides in a host cell that lacks the *lpo* membrane protein, thus releasing the peptides present in the periplasmic space outside the cells. When the producer cells are grown into colonies on a solid substrate, it leads to accumulation of the recombinant peptides around the colony and allows for easy library screening. The screening assay used in this study was a modified version of the standard agar diffusion method as previously described [20]. Library-transformed *E. coli* colonies are overlaid with a tester strain and incubated overnight to allow for peptide expression and tester strain growth. Next day, the plates are inspected for the formation of clear growth inhibition zones around *E. coli* colonies, which is an indication of active AMP production by the host colony.

#### Step 5: Sequencing of the positive clones to identify AMPs

To identify AMP sequences responsible for activity, *E. coli* colonies that are in the center of clear zones are selected and either cultured individually (for Sanger-sequencing) or grouped together based on the size of the inhibition zone (for high-throughput sequencing). Their plasmids are extracted and the peptide sequences are identified by DNA sequencing and *in silico* translation.

### Application to Discovery of Novel Plantaricin-423 Derivatives

#### Peptide and oligonucleotide library design

Based on the fact that the C-terminal region of Class IIa bacteriocins is much more diverse compared to their N-terminal region and believed to be responsible for antimicrobial activity [32], only the C-terminal region of Pln-423 was mutated in this study. A single mutation was introduced at each position, starting at the 18th amino acid, by replacing the wild-type residue with a random amino acid selected from each of six amino acid groups (positive/hydrophilic, negative/hydrophilic, polar/hydrophilic, hydrophobic, small/aliphatic, and structural) shown in Table S1. A second mutation was introduced at each remaining position again with one amino acid selected from the same groups. Single and double random deletions were also introduced at the same region of the wild-type peptide. Thus, one set of single and double mutations and one set of single and double deletions resulted in total of 12,208 unique sequences in the library (Data File S1). Each oligonucleotide sequence contained two 20mer primer-binding regions with two restriction enzyme sites, HindIII and EcoRI, and a stop codon.

#### Screening of E. coli library for novel Pln-423 variants

For the construction of Pln-423 mutant library in *E. coli*, the expression system that consists of a periplasmic-leaky *E. coli* strain JE5505 and the expression plasmid pFLAG-CTS was employed for direct screening of peptide activities. It should be noted here that this plasmid contains *ompA* secretion signal sequence and cleavage of this sequence results in a serine residue at the N-terminal of all mature peptides. A total of 1.0×10^5^ colonies, approximately 8-fold coverage of the library, were screened against *Listeria innocua* 33090 in five separate screening experiments. *L. innocua* was previously deemed as a suitable indicator for pathogenic *L. monocytogenes* displaying similar bacteriocin sensitivity [33], therefore it was used as a surrogate strain throughout this study. The selection process involved two criteria; 1) the size of the each inhibition zone was compared to that of wild-type Pln-423 as a correlation to anti-listerial activity level, 2) when several colonies formed inhibition zones that are very similar in size and character, only a few from each group were selected. The colonies were pooled into three groups based on their activities, giving 43 clones in a higher activity group (H), 81 clones in an equal activity group (E), and 241 clones in a lower activity group (L). Their plasmids were extracted from each activity group and analyzed by both Sanger and 454 high-throughput sequencing to identify peptide sequences.

#### Identification of Pln-423 variants by sanger sequencing

**Figure 2:**
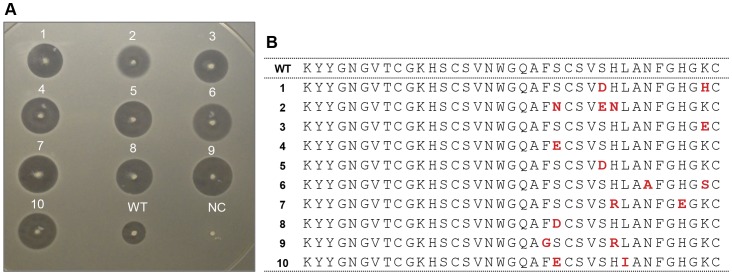
Activities and amino acid sequences of ten Pln-423 mutants tested against *L. innocua* 33090. (**A**) Clear zones indicate growth inhibition of the tester strain due to presence of active peptides produced by host colonies in the center. Wild-type Pln-423 (WT) and NC (negative control with no AMP expression) was included as controls. (**B**) Corresponding amino acid sequences of the mutants tested in A (mutations are shown in red).

Ten clones that showed the highest apparent activity on library screening plates were recovered and first analyzed by Sanger sequencing (Figure 2-B). These clones were also re-tested by colony overlay assay to compare their activities to wild-type Pln-423 based on the size of the inhibition zone around each clone (Figure 2-A). Based on this assay, all ten plantaricin mutants formed larger inhibition zones (10.3±0.5 to 11.4±0.3 mm zone diameter) compared to wild-type Pln-423 (6.2±0.2 mm) against *L. innocua* 33090. Out of those ten, three clones have a single mutation and seven clones have two mutations and the remaining single clone has three mutations. Sequence-comparison with the original Pln-423 library revealed that three of these peptides were not present in the input library. Positions like Ser23, Ser27, His28, and Lys36 were commonly mutated with similar amino acids indicating their potential role on higher peptide activity.

#### Identification of Pln-423 variants by 454 high-throughput sequencing

To demonstrate how high-throughput sequencing can be utilized to generate data on sequence-related activity of a peptide of interest, a total of 365 colonies (including ten clones that were also Sanger-sequenced) were sequenced on a 454 GS Junior. Each amplicon library was tagged with a unique multiplex identifier (MID) sequence for tracking after sequencing.

A single sequencing run generated 140,562 reads (27,989 reads in group-L, 50,592 reads in group-E and 61,981 reads in group-H) from a PCR amplicon library that was prepared by collection of three templates at equal concentrations. The reads in each group were sorted based on their length and sequence, and then the number of occurrences for each unique full-length sequence (108 to 114 bp coding region preceded by a 20 bp primer site) was determined. The majority of the sequences in each group occurred only once or twice indicating a high presence of sequencing errors (such as miscalls, overcalls, and undercalls). Considering that the peptides in our library contain two mutations at most, which means that they are 95.7% (dual mutations due to 6-base change) to 99.2% (single mutation due to one base change) identical at DNA level, discriminating between a real mutation and a sequencing error is quite challenging, thus, requires in-depth sequence analysis.

For the scope of this study, only the full-length sequences with a depth coverage of minimum 20 (determined by plotting the number of occurrence for each read versus the number of unique sequences, see Figure S1) were used for data analysis. These selected sequences were translated into amino acid sequences which yielded 149 peptides in group-L, 50 peptides in group-E, and 29 peptides in group-H. After comparing these sequences to the input Pln-423 mutant library, we determined that 118 out of 149 peptides in group-L, 40 out of 50 peptides in group-E and 25 out of 29 peptides in group-H were originated from the input library. We also observed that several peptides were present in more than one activity group; three peptides in group-L and H, six peptides in group-L and E, and six peptides in group-E and H (summarized in Table 1). Three of the peptides belonging to multiple groups had also been identified by Sanger-sequencing due to their higher anti-listerial activity on screening plates (Pln-4, 8 and 10 shown on Figure 2-A). All of the remaining sequences, although not present in the original library, contain one to four mutations at their C-terminal region (except two peptides with a mutation at the N-terminal) that are most likely introduced by errors occurring during emulsion PCR or oligonucleotide synthesis (discussed in more detail in the following section). See Data File S2 for a complete list of sequences.

**Table 1:** Summary of 454 high-throughput sequencing data analysis.

				No of peptides found in two groups		
	Group H	Group E	Group L	Groups H+E	Groups E+L	Groups H+L
No of input clones	43	81	241	–	–	–
No of output clones	29	50	149	–	–	–
No of library hits	16	28	109	6	6	3

The data obtained from 454 high-throughput sequencing showed that certain positions of Pln-423, such as Ser23, Ser25, and Ser27, His28 and His34, and Lys36, were highly mutated (18–24% of all sequences) in all activity groups. When the mutations at serine residues were closely analyzed, we observed that these residues were commonly substituted with negatively charged and/or hydrophilic amino acids (particularly aspartic acid and glutamic acid) in both group-E and group-H. However, the same positions in group-L peptides were often replaced with hydrophobic amino acids such as alanine, valine, and histidine. In addition, cysteine, proline, and tryptophan substitutions were present in a total of 21 peptides in group-L indicating detrimental effects of these mutations on peptide activity. On the other hand, while there were no mutations present at Gln20, Val26, and Ala21 in group-H peptides, these positions were highly mutated in group-L peptides. Lastly, we did not detect any mutations at positions Cys24, Gly33, and Cys37 in any of the peptides across all activity groups.

#### Determination of minimum inhibitory concentration (MIC) of selected Pln-423 variants

The MIC values were determined by the method previously described by Mills et al. [34] and Gravesen et al. [35] as the lowest concentration that produced a visible inhibition zone after 18 h of incubation at 37°C. Synthetic samples of wild-type Pln-423, two variants Pln-S23E and Pln-H28R/H34E from this study (Pln-4 and Pln-7 on Fig. 2), and another high-activity variant Pln-S27D/K36N (discovered during a pilot study, data not shown) were tested on agar plates at varying concentrations. The MIC values against *L. innocua* were determined as 0.075 µM for Pln-WT, 0.037 µM for Pln-H28R/H34E and Pln-S27D/K36N, and 1 µM for Pln-S23E (Table 2).

**Table 2:** Antimicrobial activity of wild-type and mutant plantaricin peptides against *Listeria innocua* 33090.

	Antimicrobial activity	
	MIC (µM)	Inhibition zone diam. (mm) (2.5µM peptide)
**PLN-WT**	0.075	14.3
**PLN-H28R/H34E**	0.037	18.6
**PLN-S27D/K36N**	0.037	19.1
**PLN-S23E**	1.00	ND

ND; not determined.

The MIC determined for Pln-WT agrees with literature which was a previously reported value of 0.1 µM (0.4 µg/ml) for recombinant Pln-423 [34]. The MIC values calculated for synthetic Pln-H28R/H34E and Pln-S27D/K36N also agree with the observed activity of their recombinant constructs tested with colony-overlay assay during screening. However, despite the fact that we continuously detected larger zone formation with recombinant Pln-S23E, the synthetic version of this variant showed an unexpectedly high MIC value compared to Pln-WT and other two variants.

We also employed a simple mathematical model based on Fick’s law of diffusion in three dimensions and the experimental data for the thickness of the agar and diameter of the inhibition zones (with the assumption that expression levels are and diffusion coefficients are comparable) which predicts the activity ratio between 1.5 and 1.9 times for an inhibition zone diameter ratio of 2. This finding is in good agreement with the MIC values obtained with the synthetic peptides.

## Discussion

A novel methodology for the construction and screening of custom AMP libraries has been developed in this study by expressing oligonucleotide-encoded peptides in bacterial host cells. We have successfully validated this method by generating a library coding for Pln-423 mutants in a prokaryotic host *Escherichia coli* and were able to identify more than twenty potential novel variants with enhanced specific activities against *Listeria innocua* 33090. The data generated in this study can form the basis for the construction of secondary libraries to further optimize the anti-listerial activity of Pln-423 derivatives or broaden the range of their activity against other targets.

Based on our results both from Sanger-sequencing and 454 high-throughput sequencing, certain positions of Pln-423 such as Lys36, His28, and all C-terminal serine residues were identified to be critical since the peptide activity was dramatically altered based on the type of mutations introduced at these positions. We confirmed the higher activities of mutants carrying S27D/K26N and H28R/H34E and determined their MICs against *L. innocua* as to be 2-fold lower than wild-type Pln-423. On the other hand, another variant, Pln-S23E, which was detected both in group-H and group-L by 454 sequencing, produced a 13-fold higher MIC compared to Pln-WT when synthetic peptides were tested. Although this clone was found to be highly active in numerous colony-overlay experiments, the inconsistency observed with peptide’s activity can be due to the difference in expression levels of the recombinant peptide from two separate clones or issues with the solubility of the synthetic peptide. The presence of a candidate in different apparent activity groups (group-H and group-L for Pln-S23E) could potentially be an indicator of a false positive clone. In addition, as discussed below, we have identified the wild-type Pln-423 in all three expression groups, demonstrating that clone to clone variability of expression level can be a significant source of false-positive clones. Variable solubility and stability of this peptide in different assay conditions (colony secretion versus MIC) could also explain these results. All together, these findings validate our proposed AMP discovery pipeline and stress the need for independent validation of positive candidates by MIC studies.

There are several critical steps in this process. To maintain library complexity, both emulsion PCRs (library amplification for cloning and for pyrosequencing) should be carefully designed regarding their initial template concentration. Due to high similarity between library sequences, there is a significant degree of cross-recombination events occurring during amplification within emulsion droplets when there is more than one template fragment present per droplet. Therefore, we recommend using a limited amount of template relative to the number of droplets in the emulsion. We also suggest that a preliminary experiment be performed by cloning an aliquot of the PCR products and sequencing several clones to verify library integrity.

Screening of the library was performed by a simple colony overlay assay that relies on growth inhibition of the indicator strain due to active peptides produced by the host colony. It should be noted here that the assessment of apparent activities based on the size of the inhibition zones is only for comparison purposes. Although it is a good indication of activity and commonly used as a primary screen to detect active antimicrobial compounds, we have demonstrated that false-positive clones with reduced MIC compared to wild-type but yet with larger inhibition zones can be selected. The activity cannot be completely inferred from the radius as the radius is also a function of production yield by the host colony, diffusion rate of the peptide as well as its solubility and stability. Therefore, as our results with synthetic plantaricin variants demonstrated, all peptides selected at this primary screen should be subjected to a secondary screen where purified samples of each peptide are analyzed individually to determine their MIC values as a direct measure of antimicrobial activity.

Our analysis of 454 GS Junior sequencing data yielded approximately 50% of the number of input clones selected at the screening; however, we were able to find all Sanger-sequenced input clones in group-H. We are confident that this analysis, although crude and results in potential false negatives, produced true positive sequences that were indeed selected at the library screening based on their activities while minimizing the detection of false positives due to sequencing errors. While the sequencing errors contributed to having unmatched peptides in each activity group, based on our Sanger-sequencing results we know for a fact that the input pool of 365 clones contained sequences that were not intentionally designed as a part of the library. These sequences were most likely generated due to synthesis errors or recombination events during first emPCR.

On the other hand, in-depth sequence analysis of unmatched peptides (excluding the ones detected by Sanger sequencing) revealed that most of these sequences partially match one or more library hits in their corresponding activity groups. It should be noted here that wild-type Pln-423 was found in all groups despite the fact that it was not included in the original library. Our previous experience with amplifying highly homologous DNA pools along with the information gathered in this study strongly suggests the occurrence of recombination events during library amplification for sequencing. Although we performed emulsion PCR for the generation of each amplicon library to minimize such errors, it is apparent from these findings that the initial template concentration was too high which possibly resulted in multiple template fragments per droplet, causing cross-recombination between fragments, resulting in extra sequences in the final amplicon library.

For this particular study, we have employed an *E. coli* expression system due to the fact that most Class II-a bacteriocins do not display activities against *E. coli*. In the case of generating mutants with host toxicity, we presumed that they were simply eliminated from the library during screening as those clones expressing toxic peptides would not grow into colonies. However, based on the activity spectrum of the AMP of interest, a variety of other engineered microbial systems can be utilized as the expression host in this approach, as well as other biological systems to study peptides for their binding affinities (by phage display) or for their cell-penetrating characteristics (by phage or plasmid display).

The work presented here enables the production of fully customized libraries containing hundreds of thousands of peptides in a very cost-effective way. As we attempted to demonstrate by small-scale library sequencing, this method can easily be adapted to screening of much larger libraries by employing a high-throughput screening tool combined with massively parallel deep sequencing. Robotic colony picking systems such as QPix by Molecular Devices and its “halo recognition” application can be adapted to recognition of growth inhibition zones and picking the center colonies in a high-throughput manner. Integration of such an automated system will eliminate the cumbersome colony-picking process by the researcher and will translate this method to a true high-throughput process capable of routinely producing and screening hundreds of thousands of AMP candidates. This will remarkably accelerate current AMP research towards developing novel therapeutics and biotechnological materials.

## Methods

### Construction of the Peptide Library

The oligonucleotide library was obtained from Mycroarray (Ann Arbor, MI). The oligonucleotides were amplified by emulsion PCR following the protocol developed by Williams et al. (2006) with some modifications. Briefly, 10 ng of the oligonucleotide library was mixed with a solution containing 100 pmoles of each primer, 6 mM MgCl_2_, 2 mM dNTPs, 0.5 g/l BSA, and 10 units of Phusion Hot Start DNA Polymerase (NEB) in a final volume of 100 µl. The PCR mix was emulsified by addition to 600 µl oil-surfactant mixture and stirring for 10 min at 1000 rpm on a magnetic stirrer in an ice-cooled glass vial. The emulsified reaction mix was dispensed in 50 µl aliquots and the amplification was performed by 30 cycles of 98°C for 15 s, 55°C for 20 s, and 72°C for 20 s. After extraction with two rounds of diethyl-ether and ethyl-acetate and agarose gel-purification, PCR products were digested with HindIII and EcoRI (NEB) and ligated into pFLAG-CTS expression vector (Sigma-Aldrich) linearized with the same enzymes. Ligation products were transformed into electrocompetent *E. coli JE5505* cells (Strain JE5505 was obtained from the Yale University *E. coli* Genetic Stock Center) and cloning was confirmed by DNA sequencing (University of Michigan Sequencing Core).

### Screening Assay for AMP Activity

The screening method used in this study was a modified version of the standard colony overlay method as previously described [20]. All the chemicals and growth media supplements were obtained from either Sigma-Aldrich or Fisher Scientific unless otherwise stated. Transformed *E. coli* JE5505 cells were diluted to 3000–5000 cfu (determined empirically prior to screening by plating serial dilutions of *E. coli* suspensions of a known absorbance and counting the colonies after incubation) in 25 ml LB soft agar (0.8%, 45°C) per screening plate and poured into 150 mm petri dishes. Once the media was solidified, they were overlaid with 10 ml LB soft agar (45°C) and incubated at 37°C for 24 h for host-colony formation. Next day, these plates were re-overlaid with 10 ml TSB soft agar containing 1 mM IPTG and the indicator strain *Listeria innocua* ATCC 33090 (diluted to OD_600_ = 0.03). After overnight incubation at 30°C, the plates were inspected for clear growth inhibition zones around host colonies. The peptide activity was verified by re-growing the positive hits on LB solid media (by picking AMP-producing *E. coli* colonies from screening plates and placing on fresh LB plates) for 4 h and overlaying with the indicator strain as explained above. Once the activity is confirmed, the plasmid DNA is extracted from the positive clones using Qiagen QIAprep Miniprep kit and sent for DNA sequencing for the identification of the peptide sequence.

### Generation of the Amplicon Library for 454 High-throughput Sequencing

A total of 365 colonies were individually selected from the screening plates using sterile toothpicks and placed on LB-ampicillin plates for short-term storage at 4°C. To ensure equal representation, each clone was grown separately in 250 µl LBA in 96-well plates at 37°C overnight. Based on the apparent activity of the clone compared to wild-type Pln-423, 75 µl of each overnight culture was pooled together in 3 groups. Plasmid DNA was extracted from each pooled culture using Qiagen QIAprep Miniprep kit and DNA concentration was determined by NanoDrop 1000 spectrophotometer. For the generation of amplicon libraries, both forward and reverse primers were designed following the guidelines provided by 454 junior system. Three forward primers (one for each pool) contained a 30-mer sequence adaptor, the sequencing key “TCAG” and a unique MID tag fused to a template-specific sequence. A common reverse primer composed of a 30-mer adaptor, the same sequencing key and a template-specific sequence was used for all 3 amplifications. Please see Table S2 for the list of all primers used in this study. The emulsion PCR was performed as described above except the initial template concentration was 10 ng of plasmid DNA for each reaction. The reaction conditions were as follow: one cycle at 98°C for 30 s, then 4 cycles at 98°C for 15 s, 52°C for 20 s, 72°C for 30 s and 26 cycles at 98°C for 15 s, 62°C for 20 s, 72°C for 30 s with a final cycle at 72°C for 5 min. The amplicons were purified on a 12% PAGE gel and quantified by Picogreen DNA Quantitation Kit (Molecular Probes). Equal concentrations of each amplicon library was combined and subjected to 454 pyrosequencing.

### Determination of Minimum Inhibitory Concentrations

MICs were determined as previously described [34], [35]. Synthetic peptides were obtained from Peptide 2.0, Inc. (Chantilly, VA) and dissolved in 0.1% acetic acid/water solution at the final concentration of 2.5 mg/ml. *Listeria innocua* 33090 was grown in TSB until the turbidity of the culture match that of McFarland standard 0.5 (∼OD_600_ = 0.11) and diluted to final concentration of 5×10^5^ cfu/ml in 20 ml TSB soft agar (1.2%) per plate. Two-fold serial dilutions (starting at 128 µg/ml) for each peptide was carried out in ultra-pure deionized water and 5 µl aliquots of each dilution were spotted on TSB plates. After the plates were incubated for 1 h at room temperature, they were placed at 37°C for overnight incubation. This experiment was performed in triplicate and the MIC was calculated as the lowest peptide concentration that formed a visible growth inhibition zone.

## Supporting Information

Figure S1 **Number of reads for each unique sequence generated by 454 GS Junior.** All unique sequences with a full length coding region (>128 bp, peptide-coding region plus primer binding site) were plotted versus their corresponding number of reads for each MID group (a). A close-up look at the plot (b) reveals the break point at about 20 reads per sequence for all MID groups. (MID 3: Group L, MID 4: Group E, MID 5: Group H).
(TIF)

Table S1 Amino acid groups used for peptide library design.
(PDF)

Table S2 Primer sequences used in this study.
(PDF)

Data File S1 PLN-423 mutant library.
(TXT)

Data File S2 List of PLN-423 variants from 454 sequencing.
(TXT)
