# Supplementary figures and images for: From Design to Screening: A New Antimicrobial Peptide Discovery Pipeline
Source: PLoS One. 2013 Mar 19;8(3):e59305. doi: 10.1371/journal.pone.0059305 (PMC3602187; doi:10.1371/journal.pone.0059305)

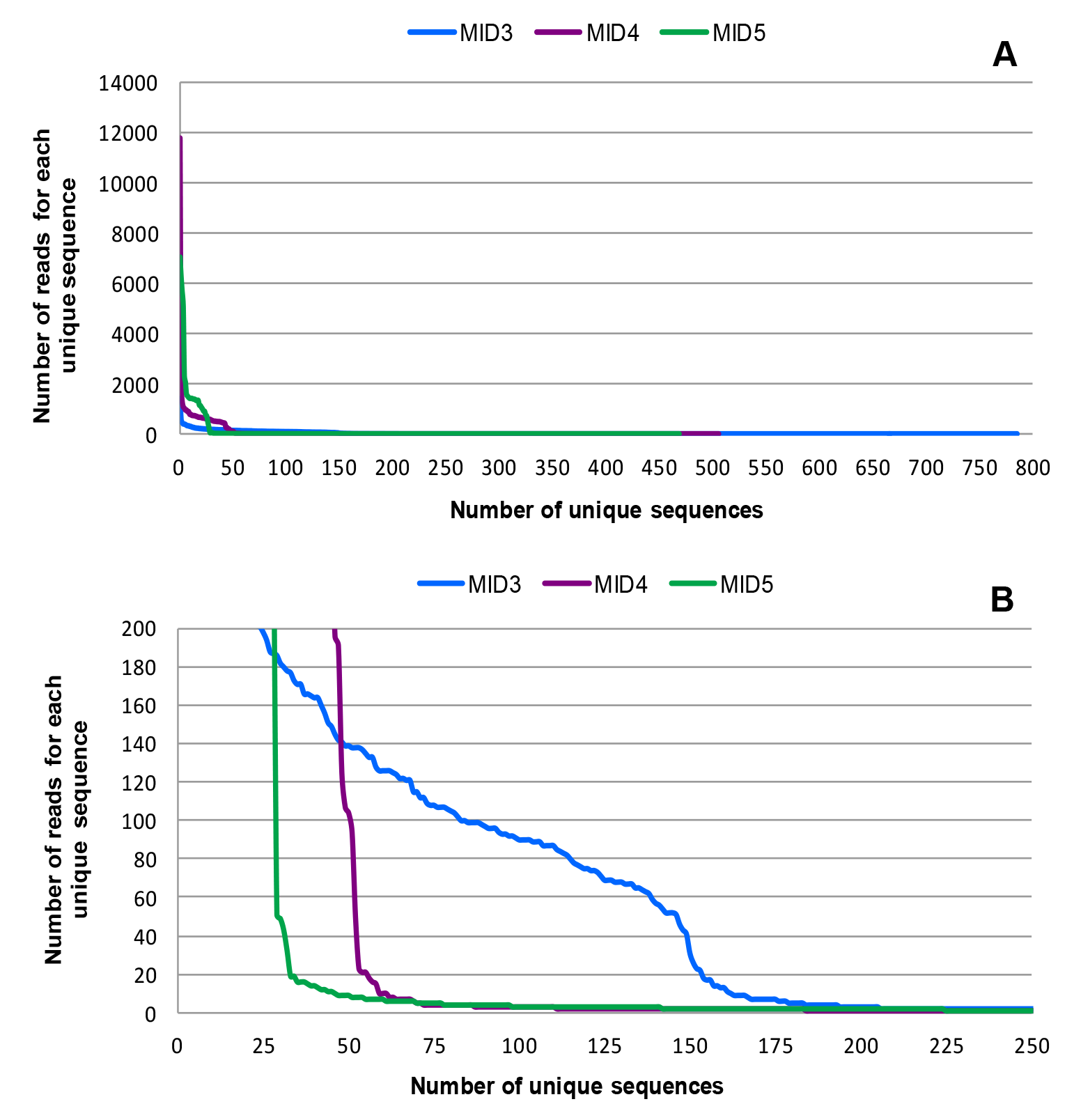

Supplement: Figure S1 — Number of reads for each unique sequence generated by 454 GS Junior. All unique sequences with a full length coding region (>128 bp, peptide-coding region plus primer binding site) were plotted versus their corresponding number of reads for each MID group (a). A close-up look at the plot (b) reveals the break point at about 20 reads per sequence for all MID groups. (MID 3: Group L, MID 4: Group E, MID 5: Group H). (TIF) [file pone.0059305.s001.tif]
