# Supplementary material for: From Design to Screening: A New Antimicrobial Peptide Discovery Pipeline
Source: PLoS One. 2013 Mar 19;8(3):e59305. doi: 10.1371/journal.pone.0059305 (PMC3602187; doi:10.1371/journal.pone.0059305)
Supplement: Table S1 — Amino acid groups used for peptide library design. (PDF) [file pone.0059305.s002.pdf]

**Table S1.** Amino acid groups used for peptide library design.

| | |
|---|---|
| **Positive and Hydrophilic** | Lysine, Arginine, Histidine |
| **Negative and Hydrophilic** | Aspartic acid, Glutamic acid |
| **Polar hydrophilic** | Serine, Threonine, Tyrosine, Asparagine, Glutamine |
| **Hydrophobic** | Valine, Leucine, Isoleucine, Methionine, Phenylalanine |
| **Aliphatic/Small** | Glycine, Alanine |
| **Structural (hydrophobic)** | Cysteine, Proline, Tryptophan |
