# Supplementary material for: From Design to Screening: A New Antimicrobial Peptide Discovery Pipeline
Source: PLoS One. 2013 Mar 19;8(3):e59305. doi: 10.1371/journal.pone.0059305 (PMC3602187; doi:10.1371/journal.pone.0059305)
Supplement: Table S2 — Primer sequences used in this study. (PDF) [file pone.0059305.s003.pdf]

Table S2. Primer sequences used in this study.

**Primer**	**Sequence (5' - 3')**
Forward Primer- Library Amp.	GCATCATAGACATAGTCAGGCATAGAGGATCC*AAGCTT*CT
Reverse Primer- Library Amp.	GCAATACGTTAGCGTTCATGGCCGTAGCTCA*GAATTC*TTA
Forward Primer-MID 3-Group L	CCATCTCATCCCTGCGTGTCTCCGACTCAG**ACACTACTCGT**CTACCGTTGCGCAAGCTTCT
Forward Primer-MID 4-Group E	CCATCTCATCCCTGCGTGTCTCCGACTCAG**ACGACACGTAT**CTACCGTTGCGCAAGCTTCT
Forward Primer-MID 5-Group H	CCATCTCATCCCTGCGTGTCTCCGACTCAG**ACGAGTAGACT**CTACCGTTGCGCAAGCTTCT
Reverse Primer-Sequencing	CCTATCCCCTGTGTGCCTTGGCAGTCTCAGATCTGGTACCCGGGAATTCTTA

Restriction sites are shown in italics and MID tags are shown in bold.
